# Trait Emotional Intelligence Questionnaire—Short Form (TEIQue-SF): A Lithuanian Validation with Preschool Teachers

**DOI:** 10.3390/jintelligence14030037

**Published:** 2026-03-02

**Authors:** Eisvina Burbaite, Ilona Tilindiene, Saulius Sukys

**Affiliations:** Department of Physical and Social Education, Lithuanian Sports University, Sporto 6, LT-44221 Kaunas, Lithuania; eisvina.burbaite@lsu.lt (E.B.); saulius.sukys@lsu.lt (S.S.)

**Keywords:** trait emotional intelligence, validation, TEIQue-SF, preschool teachers

## Abstract

Background. The present study aimed to examine the psychometric properties of the Lithuanian adaptation of the TEIQue-SF. Methods. The analyses were performed using a sample of 199 preschool teachers (100% women; mean age = 46.70, SD = 11.70 years, age range = 21–69 years) from across Lithuania. The Teacher Subjective Wellbeing Questionnaire was also administered as an external validation measure. The reliability of the TEIQue-SF was assessed by Cronbach’s α and McDonald’s ω. Finally, we examined the relationship between sociodemographic characteristics and global trait emotional intelligence. Results. Confirmatory factor analysis of the Lithuanian TEIQue-SF supported the one-factor structure of trait emotional intelligence (CFI = 0.99, TLI = 0.98, IFI = 0.99, RMSEA = 0.04, SRMR = 0.02). Good internal consistency was observed for global trait emotional intelligence (α = 0.85, ω = 0.84). Global trait emotional intelligence was significantly and positively associated with the teacher general well-being (β = 0.28), school connectedness (β = 0.26), and teaching efficacy (β = 0.28). Age was the only sociodemographic indicator positively related to global trait EI (β = 0.26). Conclusions. Our research showed that the Lithuanian version of the TEIQue-SF is a valid and reliable instrument to measure trait emotional intelligence and can be recommended for research and practical use.

## 1. Introduction

Emotional intelligence (EI) was first defined as a multidimensional psychological construct describing an individual’s ability to recognize, understand, use, and regulate emotions to promote emotional and intellectual growth (Mayer et al., 1990). In contrast to ability-based conceptualizations, trait theory emphasizes individuals’ emotional self-perceptions rather than maximal performance. From trait theory perspective, EI is conceptualized as a constellation of emotional self-perceptions, understood as a set of personality traits related to emotional functioning and self-perception in the emotional domain (Petrides & Furnham, 2001; Petrides et al., 2007). These emotional self-perceptions reflect individuals’ beliefs about their ability to understand, regulate, and utilize their own emotions as well as those of others. Empirical research has demonstrated that such self-perception exerts a significant influence on a wide range of life domains, including psychological functioning, interpersonal relationships, and well-being (Petrides et al., 2018).

Over the past few decades, EI has become a prominent topic in psychological and educational research due to its significant associations with psychological well-being (Chen et al., 2016; Larguinho et al., 2025; Llamas-Díaz et al., 2022; Urquijo et al., 2016), academic and professional achievement (Caballero-García & Ruiz, 2025; Gkintoni et al., 2024; Sharma & Tiwari, 2024), interpersonal relationships (Lopes et al., 2005; Schutte et al., 1998), and mental health outcomes (Schutte et al., 2007). At both the theoretical and applied levels, EI is therefore considered a crucial factor in effective emotional information processing, adaptive behavior across social contexts, psychological well-being, and successful professional and personal functioning.

In the educational context, the EI construct has attracted increasing scholarly attention. Empirical evidence consistently demonstrates that higher levels of teachers’ EI are associated with greater psychological and subjective well-being, higher job and life satisfaction, improved work performance, and enhancing teaching effectiveness (Abebe & Devinder, 2023; Fu et al., 2021; Hassan, 2019; Kaur et al., 2019; Li et al., 2018; Naderi Arani, 2012; Salavera & Urbón, 2024; Sekreter, 2019; Siddique et al., 2020; Soanes & Sungoh, 2019; Sökmen & Sarikaya, 2022), while simultaneously reducing the risk of burnout (Fiorilli et al., 2019; Puertas Molero et al., 2019). In early childhood education, teachers play a particularly important role in fostering children’s EI, which is considered one of the core goals of contemporary education (Chung & Han, 2024). As social-emotional skills grounded in EI are essential for functioning in an increasingly complex and dynamic world (Kankaraš & Suarez-Alvarez, 2019), research attention has increasingly focused on teachers’ EI interventions programs and valid measurement instruments (Wang et al., 2025).

The scientific literature distinguishes three main theoretical approaches to conceptualizing and measuring EI. The first, ability EI model, which conceptualizes EI as a form of cognitive ability involved in the processing of emotional information (Salovey & Mayer, 1990). The second is the mixed model, encompassing theories such as Emotional Competency theory (Goleman, 1995; Goleman & Boyatzis, 2017), which defines EI as a set of learned skills, competencies, and traits highlighting the understanding and comprehensive application of emotional knowledge and skills in various social areas, and the Emotional–Social Intelligence theory (Bar-On, 1997), which views EI as a combination of personal and interpersonal emotional–social competencies and skills, and facilitating mechanisms that operate jointly. The third approach is the trait EI model, which conceptualizes EI as a combination of personality traits related to self-esteem, self-control, tolerance, empathy, and respect for self and others (Petrides & Furnham, 2001). Within this framework, EI reflects a person’s ability to understand and control their emotions in order to develop their personality, adapt to various life situations, and be able to cope with stress. Empirical studies have confirmed that these self-perceptions influence virtually all aspects of our lives (Petrides et al., 2018). This construct could be better described as emotional self-efficacy, rather than as intellectual, ability, or competence aspects (Petrides et al., 2016).

Although ability-based, competency-based, and trait EI theories share a common goal of conceptualizing EI as a psychological construct that explains individual differences in emotional functioning, the role of emotions in psychological and social functioning, and the development of empirically grounded measurement approaches, they differ in their underlying theoretical assumptions and assessments methods. Based on the ability EI model, Mayer et al. (2002) developed the Mayer–Salovey–Caruso Emotional Intelligence Test (MSCEIT). The Emotional Quotient Inventory (EQ-i) (Bar-On, 1997) was created to assess emotional–social intelligence. Based on the EI competency model, instruments such as the ECI, ECI-2 (Emotional Competency Inventory), and the ESCI (Emotional and Social Competency Inventory) were developed (Boyatzis et al., 2000). One of the most widely used personality-based EI assessment tools worldwide is the Trait Emotional Intelligence Questionnaire (TEIQue) (Petrides, 2009).

The TEIQue is a reliable and psychometrically validated instrument designed to comprehensively assess the domains and factors of trait EI (Petrides, 2009). The full version of the instrument (TEIQue) consists of 153 items measuring 15 EI facets and four main EI trait factors: well-being, self-control, emotionality, and sociability (Petrides, 2009; Petrides & Furnham, 2003). Several versions of the TEIQue have been developed, including the TEIQue–AF (Trait Emotional Intelligence Questionnaire–Adolescent) for adolescents aged 13–17 years (Petrides, 2009), and the TEIQue–CF (Trait Emotional Intelligence Questionnaire–Child Form) to assess personality aspects related to emotions in children aged 8–12 years (Mavroveli et al., 2008). To reduce testing time and respondent burden, the short form TEIQue-SF (Trait Emotional Intelligence Questionnaire–Short Form) was developed, consisting of 30 items measuring global trait EI (Cooper & Petrides, 2010; Petrides, 2009). However, it is noted that the reliability of the four factors may be lower than that of the global EI score (Petrides, 2009).

It should be noted that different methodological approaches have been applied when validating the TEIQue-SF. For example, in Europe (specifically in Spain), confirmatory factor analysis was used to test a one-factor model in which the four trait EI factors load onto a global trait EI factor (Laborde et al., 2016; Merino-Tejedor et al., 2018; Szczygieł et al., 2015). Similar approaches were used in adapting the Chinese (Feher et al., 2019) and Arabic (Al-Dassean, 2023) versions of the TEIQue-SF. In contrast, validations of the German (Jacobs et al., 2015) and Turkish (Deniz et al., 2013) versions tested higher-order, four factor models. The Japanese adaptation applied only explanatory factor analysis (Abe et al., 2012). In other studies, outside Europe, specifically by adapting the Chilean (Pérez-Díaz & Petrides, 2021), Azerbaijani (Ismayilova, 2025), and Brazilian (Perazzo et al., 2021) versions, structure of the scale was tested using Structural Equation Modeling (SEM). Despite the existence of different methodological approaches, there was intention to evaluate whether cross-cultural differences exist in the interpretation of the TEIQue-SF (Feher et al., 2019; Perazzo et al., 2021). Findings showed that the TEIQue-SF can be interpreted as estimates of the same similar underlying construct. Recent meta-analysis conducted by Orhan (2024) showed that the TEIQue-SF has been adapted in over 15 languages and the findings also showed that this measure demonstrated construct stability and validity across cultural contexts (Orhan, 2024).

Research practice shows that in Lithuania most EI studies have employed tools based on the ability or mixed models (e.g., Schutte Self-Reported Inventory (SSRI, Schutte et al., 1998); the youth version of EQ-i (EQ-i:YV, Bar-On & James, 2000). Only few studies in Lithuania have applied a trait-based approach. Garbenis (2021) used the TEIQue-SF among special education teachers and Naudužienė and Zuzevičiūtė (2021) investigating the relationship between EI components and emotional well-being among Lithuanian university students using 67 items from the TEIQue. The factor analysis applied in the study identified factors related to emotional well-being, self-control, emotionality, sociability, and one independent factor. Although the overall reliability of the scale used was good (Cronbach’s alpha 0.74) (Naudužienė & Zuzevičiūtė, 2021). However, the adaptation and validation of the TEIQue instrument in both studies were not reported in the peer-reviewed publications.

In summary, few studies have been conducted at the national level in Lithuania, and questions remain about the validity and reliability of existing instruments within this context. In particular, there is especially a lack of studies assessing trait EI using standardized measures such as the TEIQue-SF. To ensure the reliability and cross-cultural comparability of research, it is essential that measurement tools function consistently across linguistic and cultural contexts. Previous validation studies indicate that the TEIQue-SF is an easy-to-use, not time-consuming, valid, and reliable measure of trait EI. Seeking to supplement the database on EI, in this study we aimed to validate the TEIQue-SF using a Lithuanian-speaking sample. Specifically, we seek to:

1. Examine the structural validity of the TEIQue-SF, following the Spanish model (Feher et al., 2019; Laborde et al., 2016; Merino-Tejedor et al., 2018), which has been applied not only in Europe, but also in other countries outside Europe (Al-Dassean, 2023; Feher et al., 2019) by testing a one-factor structure with four trait EI factors (represented as indicators) loading onto a global EI.
2. Assess the reliability of the TEIQue-SF. In most previous studies, the reliability of the TEIQue-SF has been assessed using Cronbach’s α (Orhan, 2024). Despite its widespread use, Cronbach alpha has recently been criticized as a method that overestimates reliability. Given growing evidence that McDonald‘s ω may provide a more appropriate measure of internal consistency (Dunn et al., 2013; Hayes & Coutts, 2020), in this study we assess reliability using both Cronbach’s α and McDonald’s ω.
3. Assess the criterion-related validity by examining the relationship between trait EI and teachers’ subjective well-being at school, hypothesizing that higher trait EI will be positively associated with subjective well-being, consistent with prior findings linking EI to job satisfaction (Miao et al., 2017; Sánchez-Álvarez et al., 2015) and teaching efficacy (Ruhela & Mishra, 2023; Wu et al., 2019).

## 2. Materials and Methods

### 2.1. Study Participants

When planning the study, the required number of participants was also considered. In our study, the sample size was determined in accordance with existing recommendations for the number of participants needed when applying confirmatory factor analysis, which we used to examine the structural validity of the TEIQue-SF. There are various recommendations regarding the minimum sample size required for confirmatory factor analysis. Comrey and Lee (1992) state that a sample size of 500 or more is very good. Although some authors identify 100 participants as an absolute minimum (Kline, 1994), a sample size of at least 200 is more commonly recommended as sufficient (Jackson et al., 2013; Mundfrom et al., 2005). Monte Carlo simulation studies further indicate that samples of approximately 200 participants are often adequate for confirmatory factor analysis (Wolf et al., 2013). It is worth noting that Comrey and Lee (1992) also describe a sample size of 200 as fair. Additionally, recommended sample size may vary depending on data characteristics, including distributional properties (normal vs. non-normal) (Muthén & Muthén, 2002), the number of observed variables, and the number of factors specified in the model (Mundfrom et al., 2005). Models with fewer variables and fewer factors generally require smaller samples. Given that the present study aimed to test the TEIQue-SF as a one-factor model, we defined the minimum number of participants in our study as 200.

The present study included a sample of preschool teachers from Lithuania. The teachers’ sample (N = 202, 100% women) was recruited from 16 preschool educational institutions across the country. However, after excluding incomplete or incorrectly completed questionnaires, the study used data from 199 teachers (all female). The participants’ ages ranged from 21 to 69 years (M = 46.66, SD = 11.70). Most teachers are married and have higher education, half have more than 15 years of teaching experience, and most work in state educational institutions and have different teaching qualifications (Table 1).

### 2.2. Translation and Cross-Cultural Adaptation

After obtaining permission to use the TEIQue-SF from the author, this measure was translated following the recommended steps for cross-cultural adaptation (Beaton et al., 2000; Hambleton & Lee, 2013). First, the questionnaire was translated into Lithuanian by three independent translators, each holding academic degrees in social sciences, educational sciences, and linguistics, with at least five years of professional experience. Second, the first synthesized version of the TEIQue-SF was done. Third, backward translation of the synthesized version into English by two independent translators to ensure the equivalence of meaning for each item. Subsequently, the panel group of specialists and the authors of this study compared Lithuania, back translation, and the original English version to produce the second synthesized Lithuanian version. Finally, the pilot study was done after that final version of the Lithuanian version of the TEIQue-SF was approved.

### 2.3. Measures

#### 2.3.1. The Short Form of the TEIQue (TEIQue-SF)

The TEIQue-SF questionnaire consists of 30 statements that assess global trait EI (Petrides, 2001, 2009). Respondents, when answering the statements, must choose a response option from 1 (completely disagree) to 7 (completely agree). It is important to note that some statements are reverse-coded during data analysis. The overall scale score is calculated by summing the responses to all statements and dividing by the number of statements. Although the short form of this questionnaire is primarily designed to assess overall EI, it can also be used to measure four trait EI factors: well-being (e.g., “I feel that I have a number of good qualities”), self-control (e.g., “On the whole, I’m able to deal with stress”), emotionality (e.g., “I often pause and think about my feelings”), and sociability (e.g., “I can deal effectively with people”). It should be noted that statements 3, 14, 18, and 29 are used solely for assessing the general EI score (Cooper & Petrides, 2010).

#### 2.3.2. The Teacher Subjective Well-Being

The Teacher Subjective Wellbeing Questionnaire (TSWQ) (Renshaw et al., 2015) was used to assess teachers’ subjective well-being; it was adapted for Lithuanians (Gidas gerovės valdymui mokyklose, 2018). Teacher well-being is a complex and multifaceted construct with various definitions and interpretations, yet it is widely recognized as including multiple dimensions. High levels of well-being are linked to employee loyalty, stronger organizational commitment, and the use of effective teaching methods. Researchers and practitioners generally agree on viewing teacher well-being as an important psychological indicator (Fox et al., 2023). Renshaw et al. (2015) state that their goal was to develop a brief, multidimensional, and efficient instrument suitable for both scientific research and practical assessment of positive psychological functioning among teachers in the workplace. The TSWQ consists of 8 items and measures teacher well-being across two subscales: school connectedness (4 items, e.g., “I feel like I belong at this school”, “I can really be myself at this school”) and teaching efficacy (4 items, e.g., “I am a successful teacher”, “I feel like my teaching is effective and helpful”). Participants had to answer using four-point scale, ranging from almost never (1) to almost always (4). In studies, both the overall well-being score and scores for each subscale can be calculated separately.

### 2.4. Procedures

Before the study, an approval from the university research ethics committee was obtained. Data were collected via anonymous online survey from April to June 2025. To collect data from the target group, the research team sent email invitations to participate in the study, along with information about the study, to 742 leaders of preschool education institutions across the country. The invitation clearly stated the study’s aims and procedures implemented to ensure anonymity and confidentiality. Participants were informed that participation was voluntary and that they could refuse or withdraw at any time without negative consequences. Upon providing electronic informed consent, they were redirected to the online questionnaire. The study therefore relied on a voluntary, non-probability sampling approach.

### 2.5. Data Analysis

First was checked data distribution by skewness and kurtosis. Skewness values of 0-2 and kurtosis of 0–7 can be taken as a result of sufficient normality (Curran et al., 1996). The data for the global and for the subscales of the TEIQue-SF demonstrated a normal distribution as skewness and kurtosis values were lower than 1.00. Next structural validity of the TEIQue-SF was evaluated. We tested a one-factor model with the four trait EI factors loading onto global trait EI by confirmatory factor analysis using JASP. To evaluate goodness of fit we used common indexes: χ^2^ (df) statistics, the Comparative Fit Index (CFI), the Tucker–Lewis Index (TLI), the Increment Fit Index (IFI), the root mean square error of approximation (RMSEA), and standardized root mean square index (SRMR). Values of 0.05 or below for RMSEA and values of 0.08 or below for SRMR were considered good fit (Hu & Bentler, 1999). Values above 0.95 for the CFI, TLI, and IFI were considered as acceptable model fit (Hu & Bentler, 1999). Reliability of the TEIQue-Sf was assessed by Cronbach’s α and McDonald ω. Correlation between the four trait EI factors and the global trait EI were calculated. We also calculated the correlation between global trait EI, four trait factors, and teachers’ well-being at school. Criterion-related validity was investigated through linear regression. First, we examined how global trait EI is associated with teachers’ well-being at work. Next, we additionally examined relations between EI factors and teachers’ well-being.

## 3. Results

### 3.1. Confirmatory Factor Analysis of the Lithuanian TEIQue-SF

**Figure 1:**
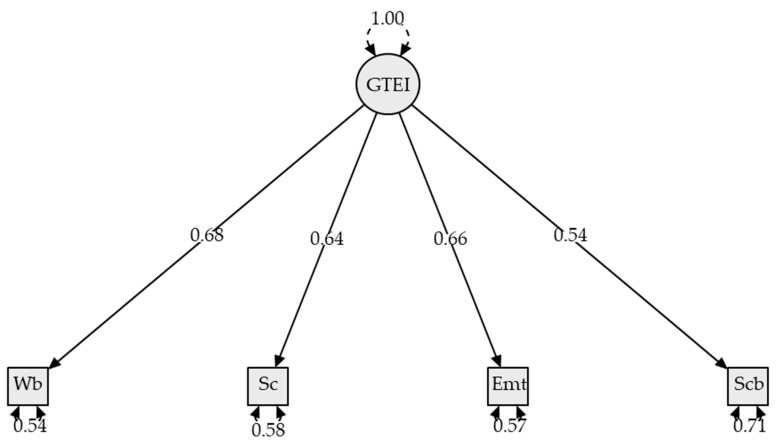
Standardized factor loadings for the TEIQue-SF Lithuanian version. Note: GTEI = Global trait emotional intelligence; Wb = Well-being; Sc = Self-control; Emt = Emotionality; Scb = Sociability.

CFA analysis was conducted to assess whether the four trait EI indicators (well-being, self-control, emotionality, sociability) fit onto the one global trait EI factor. The Lithuanian version of the TEIQue-SF showed an excellent fit providing evidence for construct validity, χ^2^(2) = 3.06, *p* = .26. CFI = 0.99, TLI = 0.98, IFI = 0.99, RMSEA = 0.04 [90% CI: 0.00; 0.15], and SRMR = 0.02. The standardized factor loadings are presented in Figure 1.

### 3.2. Descriptive Statistics, Reliability and Bivariate Correlations

Study results showed that the reliability for the global trait EI, internal consistency was good (α = 0.85, ω = 0.84. For the four factors of EI, reliability values (α as well as ω) were below 0.70, varying between 0.56 and 0.66 (Table 2).

Descriptive statistics for the four factors and global trait EI scores are presented in Table 2, with the highest mean observed for well-being and the lowest for sociability. Strong correlations were observed between global EI and well-being, self-control, and emotionality. Correlations between EI factors ranged from 0.30 to 0.45.

### 3.3. Relations of the TEIQue-SF with Teacher’s Well-Being at School

First, a correlational analysis between trait EI and well-being at school was conducted. The global trait EI score of preschool teachers was positively correlated with general well-being as well as with the two well-being subscales (Table 3). The EI factors well-being and sociability were stronger than self-control and emotionality, correlated with well-being at school.

Second, further validity of the TEIQue-SF was examined by evaluating the association between EI and preschool teachers’ well-being at school. We examined global trait EI relations with general well-being at school and separately with the two well-being subscales. The first regression analysis was significant (*F* = 17.89, *p* < .001) and revealed that trait EI positively related to the general teacher’s well-being at school (Table 4). The second regression analysis was also significant (*F* = 14.29, *p* < .001) and revealed that trait EI positively related to school connectedness. The third regression analysis also revealed that trait EI positively related to the teaching efficacy (*F* = 16.47, *p* < .001).

Although using TEIQue-SF is more appropriate to assess general trait EI, we also examined how four subscales related to the preschool teacher’s well-being at school (Table 5). In the first regression all four EI factors were entered as independent variables and general well-being as dependent variables. We found that well-being (β = 0.17, *p* = .04) and sociability (β = 0.23, *p* < .001) was significantly positively related to general teacher’s well-being at school (*F* = 5.87, *p* < .001). When examining EI factors relations with the subscales of the well-being at school we found that just well-being positively related with connectedness with the school (β = 0.17, *p* = .01) (*F* = 4.34, *p* = .01). Meanwhile, only sociability positively associated the teaching efficacy (β = 0.28, *p* < .001) (*F* = 6.45, *p* < .001).

### 3.4. Trait EI and Sociodemographic Variables

We examined how sociodemographic characteristics related to the global trait EI. By entering sociodemographic characteristics such as age, marital status, years in role, teacher qualification, education level, and status of education institution, we found age was the only indicator positively related to global trait EI (β = 0.26, 95% CI = 0.03 to 0.26, *p* = .01) (*F* = 2.23, *p* = .04). Regression models with sociodemographic and trait EI subscale well-being (*F* = 1.84, *p* = .09) and emotionality (*F* = 0.76, *p* = .60) were not significant. Trait EI subscale self-control was significantly predicted by age (β = 0.29, 95% CI = 0.10 to 0.36, *p* < .001) and teacher qualification (β = −0.17, 95% CI = −0.37 to −0.01, *p* = .04) (*F* = 6.45, *p* < .001). But the trait EI subscale sociability was significantly predicted by marital status (β = 0.17, 95% CI = 0.03 to 0.28, *p* =.02) and education level (β = 0.18, 95% CI = 0.07 to 0.47, *p* = .01) (*F* = 7.38, *p* < .001).

## 4. Discussion

The aim of this study was to test the validity of the Lithuanian version of the TEIQue-SF in a sample of preschool teachers. The results confirm that the questionnaire is structurally sound and reliable for measuring trait EI in the Lithuanian educational context. Confirmatory factor analysis showed that the four EI factors form a single higher-order construct and the model demonstrated excellent fit across all key indicators. This finding is consistent with the theoretical logic of the TEIQue-SF, according to which the short form is primarily designed to assess global trait EI rather than to analyze individual factors separately. The obtained validity results are in line with findings from studies conducted in different countries (Al-Dassean, 2023; Feher et al., 2019; Laborde et al., 2016; Merino-Tejedor et al., 2018). Importantly, however, the present results were obtained in a specific professional group—teachers—suggesting that the Lithuanian version of the TEIQue-SF adequately captures the EI construct within a professional context characterized by intensive emotional and social interaction (Garbenis, 2021).

In assessing the reliability of the Lithuanian TEIQue-SF, the global EI demonstrated very good internal consistency (α = 0.85), whereas reliability coefficients at the factor level were lower. This pattern reflects both the reliability reported by the questionnaire authors (Cooper & Petrides, 2010) and trends observed in other cross-cultural adaptations of the TEIQue-SF. A recent meta-analysis reported an average Cronbach’s alpha of 0.86 for global trait EI (Orhan, 2024), indicating that the TEIQue-Sf was specifically designed to measure global rather than factor-level EI. However, it is worth commenting on the reliability values found at the factor level. In the present study Cronbach’s alpha values ranged from 0.66 to 0.67 for well-being, self-control, and emotionality, while the lowest reliability was observed for sociability (α = 0.56). Similar results have been reported in cross-validation studies conducted with Chinese (Feher et al., 2019), Brazilian (Perazzo et al., 2021), and Spanish (Merino-Tejedor et al., 2018) samples. These findings should not be interpreted as a methodological limitation but rather as a consequence of the brevity of the subscales. Short scales typically yield lower reliability coefficients due to the limited number of items, a pattern consistently documented across countries (Orhan, 2024).

Although Cronbach‘s alpha has traditionally been used to assess reliability, recent methodological discussions have raised concern about its limitations. Therefore, we additionally calculated McDonald’s omega coefficients. These values were practically identical to the corresponding alpha coefficients, indicating robust internal consistency. Merino-Tejedor et al. (2018) similarly assessed reliability using both indices and found that alpha values higher than omega for global EI, whereas factor-level coefficients were nearly identical. This suggests that the Lithuanian version of the TEIQue-SF does not exhibit significant distortions in its internal structure.

Further validity of the Lithuanian TEIQue-SF was examined by analyzing the relationship between EI and teachers’ well-being at school. As hypothesized, EI was positively associated with teachers’ subjective well-being at school, school connectedness, and teaching efficacy. These findings are consistent with a substantial body of research demonstrating positive associations between teachers’ or lectures’ EI and organizational connectedness, job satisfaction (Naderi Arani, 2012), job performance (Abebe & Devinder, 2023; Kaur et al., 2019), subjective well-being (Hassan, 2019; Salavera & Urbón, 2024), teaching effectiveness (Sekreter, 2019; Siddique et al., 2020; Soanes & Sungoh, 2019), and reduced stress and burnout risk (Fiorilli et al., 2019; Puertas Molero et al., 2019).

In both global and Lithuanian educational contexts, teacher well-being has become an increasingly important topic due to growing professional demands and workload. Nevertheless, limited attention has been given to studying the relationships between trait EI and teachers’ well-being specifically within the school’s context. Sökmen and Sarikaya (2022) found that EI significantly predicts teachers’ job satisfaction, which in turn influences life satisfaction. Similarly, Li et al. (2018) reported a positive association between teachers’ trait EI and job performance, with job satisfaction acting as a mediating variable. Notably, research suggest that high organizational trust reduces the importance of EI in predicting work outcomes, as a supportive environment lowers the need for constant emotional regulation. Conversely, in context of low organizational trust, EI becomes more critical for maintaining job satisfaction and work effectiveness due to increased emotional demands.

The analysis of sociodemographic factors revealed that only age was significantly and positively associated with global trait EI. Although findings in this are mixed, some studies suggest that EI increases with age and follows a nonlinear developmental trajectory. EI abilities tend to be lower in younger and older ages, with peak levels observed in middle aged (Cabello et al., 2016). Bar-On (1997, 2006) argued that emotional and social maturity increases with age, leading to higher EI, while Derksen et al. (2002) found that EI generally peaks between the ages of 35 and 44 before declining. Other studies report that older adults use adaptive emotion regulation strategies more frequently and more effectively than younger adults (Dahling & Perez, 2010; Lohani & Isaacowitz, 2014). This partially aligns with our findings, as self-control factor scores were significantly associated with age and teacher qualification, the latter typically attained later in adulthood. A meta-analysis by Hodzic et al. (2018) indicated that most EI development strategies enhance emotional knowledge and understanding rather than leading to changes in thinking patterns or everyday emotional skills use. This supports the view that emotional competencies develop largely though accumulated life experiences rather than formal intervention alone (Fariselli et al., 2008; Petrides, 2010).

In our study, no significant relationship was found between teachers’ EI and teaching experience. Previous research has yielded mixed results. Valente et al. (2020) reported a negative association between teaching experience and EI, whereas Kant and Shanker (2021) found no significant differences. Valente et al. (2020) also observed a positive association between teachers’ educational level and EI, suggesting that the duration of experience alone does not necessarily lead to higher EI.

Although international research has extensively examined the relationship between EI and occupational well-being, empirical evidence within the Lithuanian teaching context remains limited. The present findings contribute to this gap and may be valuable at the organizational level by highlighting the importance of EI development for enhancing teachers’ work effectiveness. These results have practical implications for educational leadership and policy, emphasizing the need to integrate EI development with the creation of supportive, trust-based work environments within Lithuanian educational institutions.

### Limitations and Future Directions

An important limitation related to the disproportionate distribution of participants by gender: only women participated in the study. According to data from the Lithuanian Education Management Information System (2025), in the 2024–2025 academic year, 10,589 preschool teachers worked in Lithuanian preschool institutions, of whom 10,559 were women and 30 were men. This reflects the actual situation but limits the survey’s diversity. Some studies have tested the questionnaire’s reliability in male and female groups. Valente et al. (2020) found that women tend to have higher EI than male teachers, while Al-Dassean (2023) revealed that for global trait EI, well-being, self-control, and emotionality, reliability alphas for males were higher than for females, except for sociability. Although our research sample reflect the national gender distribution of preschool teachers, further research should include primary and secondary school teachers to test the TEIQue-SF for measurement invariance by gender.

Another limitation of the study concerns the limited range of research variables, which restricted a more comprehensive examination of the relationship between EI and other important aspects of teachers’ work and psychological factors. Future research is recommended to include additional variables, such as teachers’ stress levels, social support, or motivation, to better understand the role of EI and its impact on teachers’ professional activities. Including a broader set of variables would also provide stronger evidence for the questionnaire’s validity, as it would enable to use of both convergent and discriminant validity approaches in the assessment of construct validity. In summary, although this study has limitations, it provides a foundation for planning and implementing larger-scale studies in adult population.

## 5. Conclusions

The Lithuanian version of the TEIQue-SF demonstrates excellent structural validity in a sample of preschool teachers, supporting the theoretical assumption of trait EI as a global higher-order construct encompassing the interrelated dimensions of well-being, self-control, emotionality, and sociability. Although the global trait EI scale showed good internal consistency, the substantially lower reliability of the individual factors indicates that these dimensions function more coherently at the aggregate level. Accordingly, the findings reinforce the conceptualization of trait EI as a unified construct and suggest that the TEIQue-SF is best suited for assessing Global Emotional Intelligence rather than for making high-stakes decisions based on individual dimensions. This study contributes to the expansion of cross-cultural research by confirming that the TEIQue-SF is an informative and reliable instrument for measuring EI in Lithuanian education context. Furthermore, the results revealed positive and significant associations between teachers’ EI and subjective well-being at school, including connectedness to the school environment and perceived teaching effectiveness. Overall, these findings provide a strong foundation for further cross-cultural and context-specific research aimed at deepening understanding of the role of EI and its relationships with psychological and pedagogical outcomes.

## Figures and Tables

**Table 1:** Sample characteristics (N = 199).

Variable	Categories	N	%/M (SD)
Age		199	46.70 (11.70)
Age groups	21–35	40	20.1
	36–45	45	22.6
	46–55	58	29.1
	56–65	52	26.1
	65+	4	2.0
Marital status	Married or living in a partnership	134	67.3
	Single	25	12.6
	Divorced	27	13.6
	Widower/widow	13	6.5
Teaching experience (years)		199	19.05 (12.53)
Teaching experience range	≥5	44	22.1
	6–10	32	16.1
	11–15	20	10.1
	16+	103	51.8
Teacher qualification	Teacher	66	33.2
	Senior teacher	80	40.2
	Teacher methodologist	50	25.1
	Expert	3	1.5
Education	University education	143	71.9
	Lower than university education	56	28.1
Status of education institution	State	187	94.0
	Private	12	6.0

Note: N: number of participants; %: percentage; M: mean; SD: standard deviation.

**Table 2:** Descriptive statistics, reliability and correlations.

	M	SD	α/ω	Skewness/Kurtosis	1	2	3	4	5
1. Global trait EI	5.32	0.62	0.85/0.84	−0.15/−0.07					
2. Well-being	6.01	0.75	0.66/0.66	−0.94/0.98	0.70 **				
3. Self-control	5.06	0.90	0.65/0.65	−0.07/−0.21	0.75 **	0.45 **			
4. Emotionality	5.49	0.76	0.62/0.62	−0.35/−0.28	0.77 **	0.42 **	0.44 **		
5. Sociability	4.81	0.86	0.56/0.56	−0.02/−0.03	0.68 **	0.39 **	0.30 **	0.38 **	-

Note: EI: emotional intelligence; ** *p* < .01.

**Table 3:** Correlations between trait EI and teachers’ well-being at school.

	General Well-Being	Connectedness with the School	Teaching Effectiveness
Global trait EI	0.29 **	0.26 **	0.28 **
Well-being	0.26 **	0.25 **	0.24 **
Self-control	0.16 *	0.18 *	0.11
Emotionality	0.14 *	0.13	0.14 *
Sociability	0.29 **	0.22 **	0.33 **

Note: EI: emotional intelligence; * *p* < .05; ** *p* < .01.

**Table 4:** Linear regression with the teacher’s well-being at school regressed on global trait EI.

	General Well-Being			School Connectedness			Teaching Efficacy		
	R^2^ = 0.08			R^2^ = 0.07			R^2^ = 0.08		
	*SE*	β (95% CI)	*p*	*SE*	β (95% CI)	*p*	*SE*	β (95% CI)	*p*
Global trait EI	0.06	0.28 (0.12 to 0.34)	<.001	0.06	0.26 (0.11 to 0.36)	<.001	0.06	0.28 (0.12 to 0.34)	<.001

Note. SE: standard error; 95% CI: a 95% confidence interval.

**Table 5:** Linear regression with the psychological well-being at school regressed on trait EI subscales.

	*SE*	Β (95% CI)	*p*	R^2^
	**General well-being**			0.11
Well-being	0.05	0.17 (0.01 to 0.22)	.04	
Self-control	0.04	0.04 (−0.07 to 0.11)	.65	
Emotionality	0.05	−0.04 (−0.13 to 0.08)	.65	
Sociability	0.04	0.23 (0.04 to 0.22)	.01	
	**School connectedness**			0.08
Well-being	0.06	0.17 (0.01 to 0.25)	.04	
Self-control	0.05	0.08 (−0.05 to 0.15)	.33	
Emotionality	0.06	−0.04 (−0.15 to 0.09)	.65	
Sociability	0.05	0.14 (−0.01 to 0.19)	.07	
	**Teaching efficacy**			0.17
Well-being	0.05	0.11 (−0.01 to 0.20)	.09	
Self-control	0.05	−0.02 (−0.10 to 0.08)	.83	
Emotionality	0.05	−0.03 (−0.12 to 0.09)	.71	
Sociability	0.04	0.28 (0.08 to 0.25)	<.001	

Note. SE: standard error; 95% CI: a 95% confidence interval.

## Data Availability

The data utilized in this study is not publicly available due to confidentiality and privacy obligations. However, data access may be granted upon reasonable request to the corresponding author, subject to appropriate ethical approvals.

## References

[B1-jintelligence-14-00037] Abe K., Wakabayashi H., Saiki T., Kawakami C., Fujisaki K., Niwa M., Suzuki Y. (2012). Validity and reliability of the Japanese versions of the trait emotional intelligence questionnaire-short form and the jefferson scale of physician empathy. Medical Education.

[B2-jintelligence-14-00037] Abebe D. W., Devinder P. S. (2023). The relationship between emotional intelligence, job satisfaction, and job performance: Empirical evidence from public higher education institutions. European Journal of Business and Management Research.

[B3-jintelligence-14-00037] Al-Dassean K. A. (2023). Psychometric properties of the Arabic version of the Trait Emotional Intelligence Questionnaire Short Form (TEIQue-SF). Cogent Psychology.

[B4-jintelligence-14-00037] Bar-On R. (1997). The Emotional Quotient Inventory (EQ-i): A test of emotional intelligence.

[B5-jintelligence-14-00037] Bar-On R. (2006). The bar-on model of emotional-social intelligence (ESI). Psicothema.

[B6-jintelligence-14-00037] Bar-On R., James D. A. P. (2000). BarOn emotional quotient inventory: Youth version.

[B7-jintelligence-14-00037] Beaton D. E., Claire B., Francis G., Marcos B. F. (2000). Guidelines for the process of cross-cultural adaptation of self-report measures. Spine.

[B8-jintelligence-14-00037] Boyatzis R., Goleman D., Rhee K., Bar-On R., Parker J. D. A. (2000). Clustering competence in emotional intelligence: Insights from the Emotional Competence Inventory (ECI). Handbook of emotional intelligence.

[B9-jintelligence-14-00037] Caballero-García P. Á., Ruiz S. S. (2025). Emotional intelligence and its relationship with subjective well-being and academic achievement in university students. Journal of Intelligence.

[B10-jintelligence-14-00037] Cabello R., Sorrel M. A., Fernández-Pinto I., Extremera N., Fernández-Berrocal P. (2016). Age and gender differences in ability emotional intelligence in adults: A cross-sectional study. Developmental Psychology.

[B11-jintelligence-14-00037] Chen Y., Peng Y., Fang P. (2016). Emotional intelligence mediates the relationship between age and subjective well-being. The International Journal of Aging and Human Development.

[B12-jintelligence-14-00037] Chung H., Han E. (2024). How does emotional intelligence develop in early childhood?: A meta-analysis of two perspectives on emotional intelligence. Early Years.

[B13-jintelligence-14-00037] Comrey A. L., Lee H. B. (1992). A first course in factor analysis.

[B14-jintelligence-14-00037] Cooper A., Petrides K. V. (2010). A psychometric analysis of the Trait Emotional Intelligence Questionnaire–Short Form (TEIQue–SF) using item response theory. Journal of Personality Assessment.

[B15-jintelligence-14-00037] Curran P. J., West Stephen G., Finch J. F. (1996). The robustness of test statistics to nonnormality and specification error in confirmatory factor analysis. Psychological Methods.

[B16-jintelligence-14-00037] Dahling J., Perez L. A. (2010). Older worker, different actor? Linking age and emotional labor strategies. Personality and Individual Differences.

[B17-jintelligence-14-00037] Deniz M. E., Özer E., Işık E. (2013). Trait Emotional Intelligence Questionnaire–Short Form: Validity and reliability studies. Education and Science.

[B18-jintelligence-14-00037] Derksen J., Kramer I., Katzko M. (2002). Does a self-report measure for emotional intelligence assess something different than general intelligence?. Personality and Individual Differences.

[B19-jintelligence-14-00037] Dunn T. J., Baguley T., Brunsden V. (2013). From Alpha to Omega: A practical solution to the pervasive problem of internal consistency estimation. British Journal of Psychology.

[B20-jintelligence-14-00037] Education Management Information System (2025). https://www.svis.smm.lt/en/.

[B21-jintelligence-14-00037] Fariselli L., Ghini M., Freedman J. (2008). Age and emotional intelligence. White paper research on emotional intelligence.

[B22-jintelligence-14-00037] Feher A., Yan G., Saklofske D. H., Plouffe R. A., Gao Y. (2019). An investigation of the psychometric properties of the Chinese Trait Emotional Intelligence Questionnaire Short Form (Chinese TEIQue-SF). Frontiers in Psychology.

[B23-jintelligence-14-00037] Fiorilli C., Benevene P., De Stasio S., Buonomo I., Romano L., Pepe A., Addimando L. (2019). Teachers’ burnout: The role of trait emotional intelligence and social support. Frontiers in Psychology.

[B24-jintelligence-14-00037] Fox H. B., Walter H. L., Ball K. B. (2023). Methods used to evaluate teacher well-being: A systematic review. Psychology in the Schools.

[B25-jintelligence-14-00037] Fu W., Wang C., Tang W., Lu S., Wang Y. (2021). Emotional intelligence and well-being of special education teachers in China: The mediating role of work-engagement. Frontiers in Psychology.

[B26-jintelligence-14-00037] Garbenis S. (2021). Trait emotional intelligence of teachers working in special education schools. Human, Technologies and Quality of Education.

[B27-jintelligence-14-00037] (2018). Gidas gerovės valdymui mokyklose [Guide to wellbeing management in schools].

[B28-jintelligence-14-00037] Gkintoni E., Dimakos I., Nikolaou G. (2024). Cognitive insights from emotional intelligence: A systematic review of EI models in educational achievement. Emerging Science Journal.

[B29-jintelligence-14-00037] Goleman D. (1995). Emotional intelligence: Why it can matter more than IQ for character, health and lifelong achievement.

[B30-jintelligence-14-00037] Goleman D., Boyatzis R. (2017). Emotional intelligence has 12 elements. Which do you need to work on?. Harvard Business Review.

[B31-jintelligence-14-00037] Hambleton R. K., Lee M. K., Saklofske D. H., Reynolds C. R., Schwean V. (2013). Methods for translating and adapting tests to increase cross-language validity. The Oxford handbook of child psychological assessment.

[B32-jintelligence-14-00037] Hassan M. U. (2019). Emotional intelligence and subjective well being: Exploration of teachers’ burning dilemma. Problems of Psychology in the 21st Century.

[B33-jintelligence-14-00037] Hayes A. F., Coutts J. J. (2020). Use Omega rather than Cronbach’s Alpha for estimating reliability. But…. Communication Methods and Measures.

[B34-jintelligence-14-00037] Hodzic S., Scharfen J., Ripoll P., Holling H., Zenasni F. (2018). How efficient are emotional intelligence trainings: A meta-analysis. Emotion Review.

[B35-jintelligence-14-00037] Hu L., Bentler P. M. (1999). Cutoff criteria for fit indexes in covariance structure analysis: Conventional criteria versus new alternatives. Structural Equation Modeling: A Multidisciplinary Journal.

[B36-jintelligence-14-00037] Ismayilova N. (2025). Psychometric evaluation of the Azerbaijani version of the Trait Emotional Intelligence Questionnaire-Short Form (TEIQue-SF). Current Psychology.

[B37-jintelligence-14-00037] Jackson D. L., Voth J., Frey M. P. (2013). A note on sample size and solution propriety for confirmatory factor analytic models. Structural Equation Modeling: A Multidisciplinary Journal.

[B38-jintelligence-14-00037] Jacobs I., Sim C. W., Zimmermann J. (2015). The German TEIQue-SF: Factorial structure and relations to agentic and communal traits and mental health. Personality and Individual Differences.

[B39-jintelligence-14-00037] Kankaraš M., Suarez-Alvarez J. (2019). Assessment framework of the OECD study on social and emotional skills *(OECD education working papers. 207)*.

[B40-jintelligence-14-00037] Kant R., Shanker A. (2021). Relationship between emotional intelligence and burnout: An empirical investigation of teacher educators. International Journal of Evaluation and Research in Education (IJERE).

[B41-jintelligence-14-00037] Kaur I., Charu S., Mital K. M. (2019). The role of emotional intelligence competencies in effective teaching and teacher’s performance in higher education. Higher Education for the Future.

[B42-jintelligence-14-00037] Kline P. (1994). Easy guide to factor analysis.

[B43-jintelligence-14-00037] Laborde S., Allen M. S., Guillén F. (2016). Construct and concurrent validity of the short- and long-form versions of the trait emotional intelligence questionnaire. Personality and Individual Differences.

[B44-jintelligence-14-00037] Larguinho M., Leal S., Lopes R. (2025). The impact of emotional intelligence on the psychological well-being of young graduates in Portugal. Psychology International.

[B45-jintelligence-14-00037] Li M., Pérez-Díaz P. A., Mao Y., Petrides K. V. (2018). A multilevel model of teachers’ job performance: Understanding the effects of trait emotional intelligence, job satisfaction, and organizational trust. Frontiers in Psychology.

[B46-jintelligence-14-00037] Llamas-Díaz D., Cabello R., Megías-Robles A., Fernández-Berrocal P. (2022). Systematic review and meta-analysis: The association between emotional intelligence and subjective well-being in adolescents. Journal of Adolescence.

[B47-jintelligence-14-00037] Lohani M., Isaacowitz D. M. (2014). Age differences in managing response to sadness elicitors using attentional deployment, positive reappraisal and suppression. Cognition and Emotion.

[B48-jintelligence-14-00037] Lopes P. N., Salovey P., Côté S., Beers M. (2005). Emotion regulation abilities and the quality of social interaction. Emotion.

[B49-jintelligence-14-00037] Mavroveli S., Petrides K. V., Shove C., Whitehead A. (2008). Investigation of the construct of trait emotional intelligence in children. European Child and Adolescent Psychiatry.

[B50-jintelligence-14-00037] Mayer J. D., DiPaolo M., Salovey P. (1990). Perceiving affective content in ambiguous visual stimuli: A component of emotional intelligence. Journal of Personality Assessment.

[B51-jintelligence-14-00037] Mayer J. D., Salovey P., Caruso D. (2002). Mayer-Salovey-Caruso Emotional Intelligence Test (MSCEIT) users manual.

[B52-jintelligence-14-00037] Merino-Tejedor E., Hontangas P. M., Petrides K. V. (2018). Career adaptability mediates the effect of trait emotional intelligence on academic engagement. Revista de Psicodidáctica (English Edition).

[B53-jintelligence-14-00037] Miao C., Humphrey R. H., Shanshan Q. (2017). A meta-analysis of emotional intelligence effects on job satisfaction mediated by job resources, and a test of moderators. Personality and Individual Differences.

[B54-jintelligence-14-00037] Mundfrom D. J., Shaw D. G., Ke T. L. (2005). Minimum sample size recommendations for conducting factor analyses. International Journal of Testing.

[B55-jintelligence-14-00037] Muthén L. K., Muthén B. O. (2002). How to use a Monte Carlo study to decide on sample size and determine power. Structural Equation Modelling.

[B56-jintelligence-14-00037] Naderi Arani N. (2012). Teachers: Emotional intelligence, job satisfaction, and organizational commitment. Journal of Workplace Learning.

[B57-jintelligence-14-00037] Naudužienė G., Zuzevičiūtė V. (2021). Emocinio intelekto ir psichologinės gerovės ryšys: Ugdymo galimybės [The relationship between emotional intelligence and psychological well-being: Educational possibilities]. Šiuolaikinės Visuomenės Ugdymo Veiksniai.

[B58-jintelligence-14-00037] Orhan A. (2024). Trait Emotional Intelligence Questionnaire Short Form (TEIQue-SF): Reliability generalization meta-analysis. Personality and Individual Differences.

[B59-jintelligence-14-00037] Perazzo M. F., Abreu L. G., Pérez-Díaz P. A., Petrides K. V., Granville-Garcia A. F., Paiva S. M. (2021). Trait Emotional Intelligence Questionnaire-Short Form: Brazilian validation and measurement invariance between the United Kingdom and Latin-American Datasets. Journal of Personality Assessment.

[B60-jintelligence-14-00037] Petrides K. V. (2001). A psychometric investigation into the construct of emotional intelligence. Unpublished doctoral dissertation.

[B61-jintelligence-14-00037] Petrides K. V., Parker J., Saklofske D., Stough C. (2009). Psychometric properties of the Trait Emotional Intelligence Questionnaire (TEIQue). Assessing emotional intelligence.

[B62-jintelligence-14-00037] Petrides K. V. (2010). Trait emotional intelligence theory. Industrial and Organizational Psychology.

[B63-jintelligence-14-00037] Petrides K. V., Furnham A. (2001). Trait emotional intelligence: Psychometric investigation with reference to established trait taxonomies. European Journal of Personality.

[B64-jintelligence-14-00037] Petrides K. V., Furnham A. (2003). Trait emotional intelligence: Behavioural validation in two studies of emotion recognition and reactivity to mood induction. European Journal of Personality.

[B65-jintelligence-14-00037] Petrides K. V., Pita R., Kokkinaki F. (2007). The location of trait emotional intelligence in personality factor space. British Journal of Psychology.

[B66-jintelligence-14-00037] Petrides K. V., Sanchez-Ruiz M. J., Siegling A. B., Saklofske D. H., Mavroveli S., Keefer K. V., Parker J. D. A., Saklofske D. H. (2018). Emotional intelligence as personality: Measurement and role of trait emotional intelligence in educational contexts. Emotional intelligence in education: Integrating research with practice.

[B67-jintelligence-14-00037] Petrides K. V., Siegling A. B., Saklofske D. H., Kumar U. (2016). Theory and measurement of trait emotional intelligence. The wiley handbook of personality assessment.

[B68-jintelligence-14-00037] Pérez-Díaz P. A., Petrides K. V. (2021). The Spanish-Chilean Trait Emotional Intelligence Questionnaire-Short Form: The adaptation and validation of the TEIQue-SF in Chile. Journal of Personality Assessment.

[B69-jintelligence-14-00037] Puertas Molero P., Zurita Ortega F., Ubago Jiménez J. L., González Valero G. (2019). Influence of emotional intelligence and burnout syndrome on teachers well-being: A systematic review. Social Sciences.

[B70-jintelligence-14-00037] Renshaw T. L., Long A. C., Cook C. R. (2015). Assessing teachers’ positive psychological functioning at work: Development and validation of the Teacher Subjective Wellbeing Questionnaire. School Psychology Quarterly.

[B71-jintelligence-14-00037] Ruhela V. S., Mishra P. (2023). The role of special educators’ emotional intelligence in self-efficacy: A review. Journal of Arts, Humanities and Social Sciences.

[B72-jintelligence-14-00037] Salavera C., Urbón E. (2024). Emotional wellbeing in teachers. Acta Psychologica.

[B73-jintelligence-14-00037] Salovey P., Mayer J. D. (1990). Emotional intelligence. Imagination, Cognition and Personality.

[B74-jintelligence-14-00037] Sánchez-Álvarez N., Extremera N., Fernández-Berrocal P. (2015). The relation between emotional intelligence and subjective well-being: A meta-analytic investigation. The Journal of Positive Psychology.

[B75-jintelligence-14-00037] Schutte N. S., Malouff J. M., Hall L. E., Haggerty D. J., Cooper J. T., Golden C. J., Dornheim L. (1998). Development and validation of a measure of emotional intelligence. Personality and Individual Differences.

[B76-jintelligence-14-00037] Schutte N. S., Malouff J. M., Thorsteinsson E. B., Bhullar N., Rooke S. E. (2007). A meta-analytic investigation of the relationship between emotional intelligence and health. Personality and Individual Differences.

[B77-jintelligence-14-00037] Sekreter G. (2019). Emotional intelligence as a vital indicator of teacher effectiveness. International Journal of Social Sciences & Educational Studies.

[B78-jintelligence-14-00037] Sharma S., Tiwari V. (2024). Does emotional intelligence contribute to career success? Evidence from a systematic literature review. Global Business and Organizational Excellence.

[B79-jintelligence-14-00037] Siddique M., Taseer N. A., Siddique M. (2020). Teachers’ emotional intelligence and teaching effectiveness: A correlational study. Ilkogretim Online—Elementary Education Online.

[B80-jintelligence-14-00037] Soanes D. G., Sungoh S. (2019). Influence of emotional intelligence on teacher effectiveness of science teachers. Psychology.

[B81-jintelligence-14-00037] Sökmen Y., Sarikaya İ. (2022). The mediating role of self-efficacy between emotional intelligence and job satisfaction of primary school teachers. European Review of Applied Psychology.

[B82-jintelligence-14-00037] Szczygieł D., Jasielska A., Wytykowska A. (2015). Psychometric properties of the Polish version of the Trait Emotional Intelligence Questionnaire-Short Form. Polish Psychological Bulletin.

[B83-jintelligence-14-00037] Urquijo I., Extremera N., Villa A. (2016). Emotional intelligence, life satisfaction, and psychological well-being in graduates: The mediating effect of perceived stress. Applied Research in Quality of Life.

[B84-jintelligence-14-00037] Valente S., Veiga-Branco A., Rebelo H., Lourenço A. A., Cristóvão A. M. (2020). The relationship between emotional intelligence ability and teacher efficacy. Universal Journal of Educational Research.

[B85-jintelligence-14-00037] Wang Y., Zai F., Zhou X. (2025). The impact of emotion regulation strategies on teachers’ well-being and positive emotions: A meta-analysis. Behavioral Sciences.

[B86-jintelligence-14-00037] Wolf E. J., Harrington K. M., Clark S. L., Miller M. W. (2013). Sample size requirements for structural equation models: An evaluation of power, bias, and solution propriety. Educational and Psychological Measurement.

[B87-jintelligence-14-00037] Wu Y., Lian K., Hong P., Liu S., Lin R. M., Lian R. (2019). Teachers’ emotional intelligence and self-efficacy: Mediating role of teaching performance. Social Behavior and Personality: An International Journal.

